# The Resistance–Amplitude–Frequency Effect of In–Liquid Quartz Crystal Microbalance

**DOI:** 10.3390/s17071476

**Published:** 2017-06-22

**Authors:** Xianhe Huang, Qingsong Bai, Qi Zhou, Jianguo Hu

**Affiliations:** School of Automation Engineering, University of Electronic Science and Technology of China, Chengdu 611731, China; baiqingsong@std.uestc.edu.cn (Q.B.); 2014050101009@std.uestc.edu.cn (Q.Z.); hujianguo@std.uestc.edu.cn (J.H.)

**Keywords:** quartz crystal microbalance (QCM), equivalent circuit, resistance-amplitude-frequency effect, in-liquid QCM

## Abstract

Due to the influence of liquid load, the equivalent resistance of in-liquid quartz crystal microbalance (QCM) increases sharply, and the quality factor and resonant frequency decreases. We found that the change in the resonant frequency of in-liquid QCM consisted of two parts: besides the frequency changes due to the mass and viscous load (which could be equivalent to motional inductance), the second part of frequency change was caused by the increase of motional resistance. The theoretical calculation and simulation proved that the increases of QCM motional resistance may indeed cause the decreases of resonant frequency, and revealed that the existence of static capacitance was the root cause of this frequency change. The second part of frequency change (due to the increases of motional resistance) was difficult to measure accurately, and may cause great error for in-liquid QCM applications. A technical method to reduce the interference caused by this effect is presented. The study contributes to the accurate determination of the frequency and amplitude change of in-liquid QCM caused by liquid load, which is significant for the QCM applications in the liquid phase.

## 1. Introduction

Quartz crystal microbalance (QCM) has been used for a long time in vacuum and gaseous environments as an ultrasensitive mass sensor, especially for film thickness monitoring [1,2], humidity sensors [3,4,5], proximity sensors [6,7], and various gas sensors [8,9,10]. A QCM device typically consists of a thin disk of AT-cut quartz crystal with circular electrodes patterned on both sides. Due to the piezoelectric properties and crystalline orientation of quartz, the alternating voltage between the electrodes excites crystal oscillating in a thickness shear mode. With varying mass attached to one side of the electrode, the QCM resonant frequency changes accordingly. The fundamental theory describing the relationship between frequency change and mass change was derived by Sauerbrey [11]; however, this theory is limited to the assumption that the mass deposition forms a thin, rigid film and that the mass sensitivity is uniform over the entire surface [12]. Since Nomura and Okuhara [13] showed that a QCM completely immersed in a liquid could also be excited to stable oscillation [14]—which marked the beginning of the QCM’s applications in liquid phase—QCM has often been applied as electrochemistry sensors [15,16,17], biosensors [18,19,20,21], and immunosensors [22,23,24,25].

**Figure 1:**
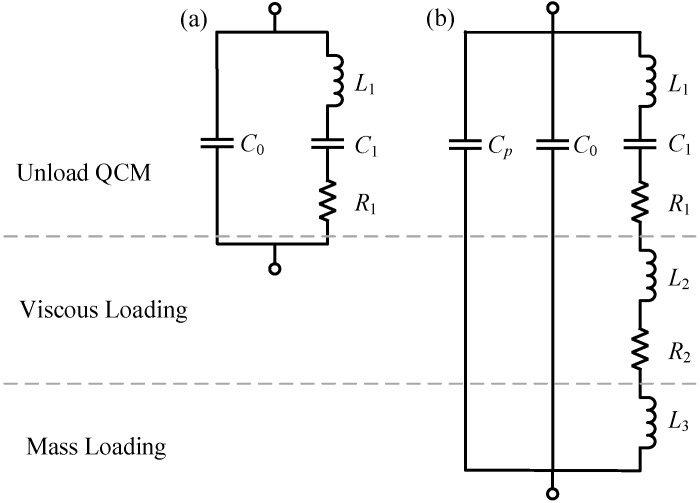
The Butterworth-Van Dyke (BVD) equivalent circuit for (**a**) an unloaded quartz crystal microbalance (QCM); and (**b**) a QCM under viscous and mass loading.

It is convenient to use a lumped-parameter equivalent circuit to characterize a QCM for a narrow range of frequencies near resonance. In such a frequency range, the circuit parameters can be considered constant. Several years before the discovery of the piezoelectric resonator, Van Dyke and Butterworth showed that any mechanically vibrating system driven by means of an electrostatic field could be represented by the Butterworth-Van Dyke (BVD) equivalent circuit [26,27]. Therefore, the BVD equivalent circuit is typically used to describe the unloaded QCM (Figure 1a). In the BVD model, $C_{0}$, $C_{1}$, $L_{1}$, and $R_{1}$ are the static capacitance, motional capacitance, motional inductance, and motional resistance for unloaded QCM, respectively.

However, in-liquid QCM applications are more important and complex, as the existence of a liquid load causes sharp increases of equivalent resistance and resonance damping, which consequently decreases the quality factor and resonant frequency. Martin et al. presented a model of an equivalent circuit for in-liquid QCM, which is shown in Figure 1b [28]. In Martin’s model, the influence caused by a liquid load was divided into mass loading and viscous loading; inductance $L_{2}$ and resistance $R_{2}$ were used to approximate the impedance of the viscous loading; inductance $L_{3}$ was used to approximate the impedance of the mass loading; and capacitance $C_{p}$ was used to express the parasitic capacitance of the test fixture [28]. In general, the increased resistance $R_{2}$ (caused by viscous loading) was much larger than $R_{1}$, which is the motional resistance of unloaded QCM. The increased inductance ($L_{2}$ and $L_{3}$) and the increased resistance ($R_{2})$ caused by viscous loading resulted in the decreases of QCM resonant frequency.

It is noteworthy that the resonant frequency f we measured in most in-liquid QCMs was the S21 minimum impedance frequency rather than the zero-phase frequency, where S21 is an S-parameter which represents the forward transmission coefficient of two-port networks. There were two reasons: (1) for practical in-liquid QCM applications, when equivalent resistance increased to a certain degree, the S21 phase curve could not reach zero (that is, the zero-phase frequency would disappear); and (2) due to the existence of scatter phase shift in circuits, the actual oscillation frequency of oscillators was the minimum impedance frequency instead of the zero-phase frequency.

## 2. Theory and Simulation

The equivalent circuit of QCM loaded with liquid could be divided into the static arm branch and the motional arm branch. The static arm branch contained $C_{0}$ and $C_{p}$, where $C_{p}$ is the parasitic capacitance arising in the test fixture; and $C_{0}$ is the static capacitance arising from the two electrodes separated by the insulating quartz and is a function of the electrodes size, shape, and configuration. Due to the piezoelectric effect of quartz crystal, the electromechanical coupling gave rise to the motional arm branch ($C_{1}$, $L_{1}$, $R_{1}$, $L_{2}$, $R_{2}$, and $L_{3}$). If we define that, the impedance of the static arm branch ($Z_{s}$) and the motional arm branch ($Z_{m}$) can be obtained:

(1) $$Z_{s}=1/j\omega C^{*}$$

(2) $$Z_{m}=R^{*}+j\omega L^{*}+1/j\omega C_{1}$$

where $C^{*}=C_{0}+C_{p}$; $R^{*}=R_{1}+R_{2}$; $L^{*}=L_{1}+L_{2}+L_{3}$; and ω=2πf. Consequently, the total impedance Z and admittance Y of the in-liquid QCM are obtained:

(3) $$Z=\frac{Z_{s}Z_{m}}{Z_{s}+Z_{m}}=\frac{1-\omega^{2}L^{*}C_{1}+j\omega R^{*}C_{1}}{-\omega^{2}R^{*}C^{*}C_{1}-j\omega^{3}L^{*}C^{*}C_{1}+j\omega \left(C^{*}+C_{1}\right)}$$

(4) $$Y=\frac{R^{*}}{{R^{*}}^{2}+{\left(\omega L^{*}-1/\omega C_{1}\right)}^{2}}+j\left[\frac{\left(\omega L^{*}-1/\omega C_{1}\right)}{{R^{*}}^{2}+{\left(\omega L^{*}-1/\omega C_{1}\right)}^{2}}+\omega C^{*}\right]$$

Therefore, the resonant frequency f is the frequency at the point of minimum impedance $Z_{min}$ (or the point of maximum admittance $Y_{max}$).

The increased inductance $L_{2}+L_{3}$ caused by mass and viscous loading is what we normally think of as the reason for change in QCM resonant frequency: since the resonant frequency $f=1/\left(2\pi \sqrt{L^{*}C_{1}}\right)$, where $L^{*}=L_{1}+L_{2}+L_{3}$ and $L_{1}\gg L_{2}+L_{3}$, and $C_{1}$ would not change for mass or viscous loading, therefore the fractional change of resonant frequency is as in Reference [28].

(5) $$\frac{\Delta f_{L}}{f}=-\frac{\Delta L}{2L^{*}}=-\frac{\left(L_{2}+L_{3}\right)}{2\left(L_{1}+L_{2}+L_{3}\right)}$$

Nevertheless, it is worth noting that increased motional resistance may also lead to resonant frequency change. Unfortunately, only the resonance damping caused by the added motional resistance has been considered, but the resonant frequency change caused by the added motional resistance had long remained unknown.

The calculated results of the equivalent circuit illustrated the resonant frequency change caused by the added motional resistance. The equivalent electronic parameters of an AT-cut 10 MHz QCM with one surface loaded by a drop of lube oil were measured. Considering that liquid loading results in great increases of resonance damping and great decreases of Q factor, the short-term frequency stability worsens significantly; therefore, we adopted a passive forward transmission measurement method with a vector network analyzer (Agilent E5062A). The radius of the oil drop was approximately 1.2 mm. The diameters of the crystal wafer and gold electrode were 8.7 and 5.1 mm, and the thicknesses of the gold electrodes and quartz crystal wafer were about 1000 Å and 0.167 mm, respectively. Due to the droplet loading, the measured resonance frequency decreased from 9.999873 MHz to 9.992959 MHz. The static capacitance $C_{0}$ of the QCM was 6.0 pF; and the parasitic capacitance $C_{p}$ formed in the test fixture was about 2.5 pF. The motional capacitance $C_{1}$ of the QCM was 27.0 fF. The motional resistance $R_{1}$ of the unload QCM and the motional resistance $R_{2}$ caused by oil loading were approximately 12 Ω and 690 Ω, respectively. The motional inductance $L^{*}=L_{1}+L_{2}+L_{3}$ of the loaded QCM was about 9.3816 mH, where $L_{1}\gg L_{2}+L_{3}$.

Table 1 shows the calculation results obtained by MATLAB software (The Math Works, Inc.) using the BVD equivalent circuit model. The resonant frequency and resonant amplitude of QCM with and without added motional resistance were calculated. It was clear that when other parameters remained constant, the motional resistance increase caused both frequency change and resonance damping simultaneously. The resonant frequency change $\Delta f_{R}$ caused by the added motional resistance was influenced by the static capacitance $C^{*}$: $\Delta f_{R}$ increased sharply along with $C^{*}$ increasing. Moreover, the resonance damping caused by the added motional resistance decreased along with $C^{*}$ increasing. That is, the smaller the static capacitance $C^{*}$, the larger the resonance damping caused by the added motional resistance.

**Figure 2:**
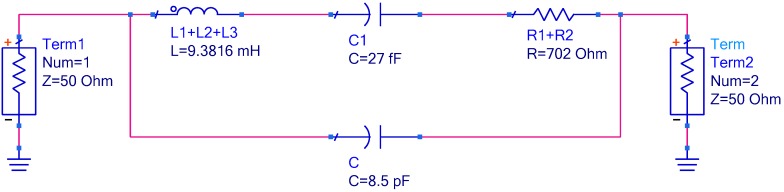
The Butterworth-Van Dyke (BVD) equivalent circuit schematic of Advanced Design System (ADS) simulation.

**Figure 3:**
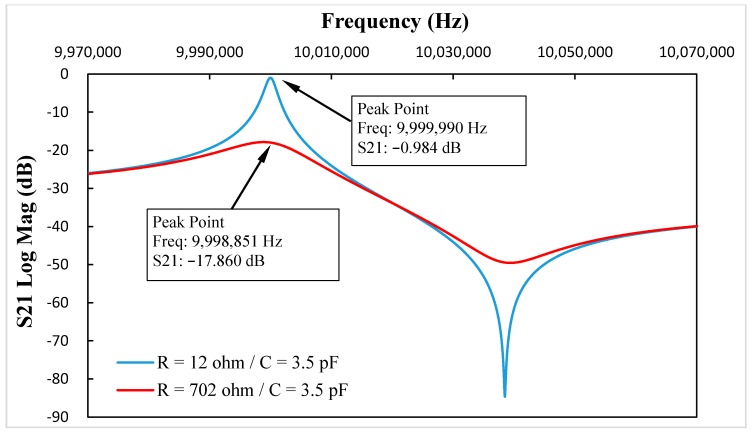
When $C^{*}$ = 3.5 pF, the S21 parameters vs. frequency curve with and without added motional resistance.

**Figure 4:**
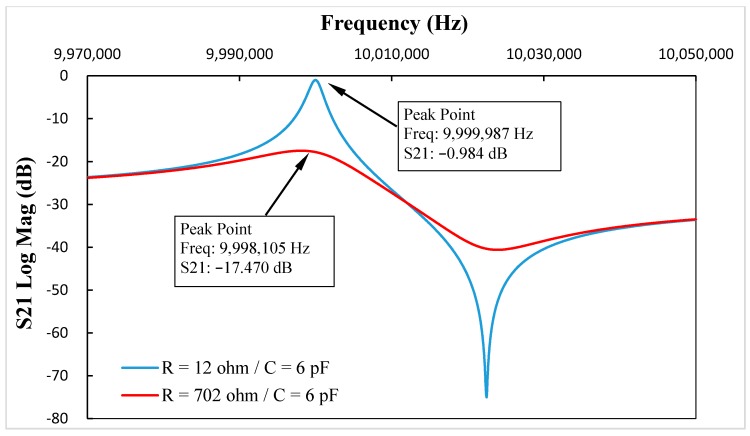
When $C^{*}$ = 6.0 pF, the S21 parameters vs. frequency curve with and without added motional resistance.

**Figure 5:**
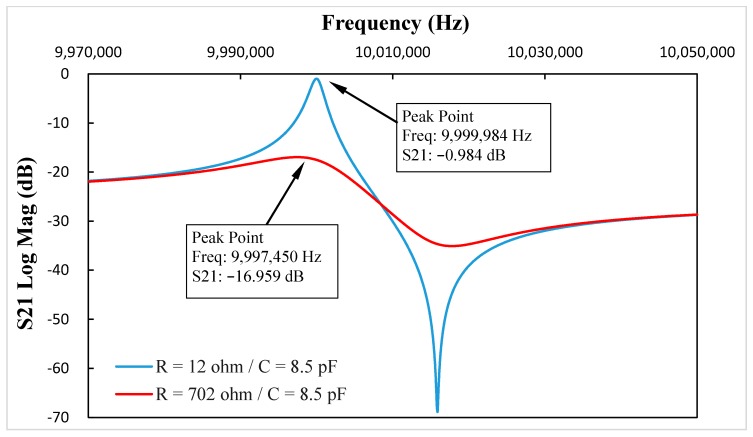
When $C^{*}$ = 8.5 pF, the S21 parameters vs. frequency curve with and without added motional resistance.

To illustrate the resistance-amplitude-frequency effect of in-liquid QCM more clearly, simulations were carried out using business software Advanced Design System (Agilent Technologies, Inc. Santa Clara, CA, USA) a professional commercial circuit simulation tool software and well suited to simulate the circuit’s S-parameters. The scheme of the BVD equivalent circuit simulation is shown in Figure 2. Using Groups 3–5 in Table 1 as instances, the S21 parameters vs. frequency curve with and without the added motional resistance are shown as Figure 3, Figure 4 and Figure 5, which reflect the same phenomenon.

By comparing the data in Table 1 and Figure 3, Figure 4 and Figure 5, the resonance damping caused by the added motional resistance was the same, and the changes in resonance frequency were different, but had the same trend. The reason leading to different resonance frequency shifts between the calculation results and the simulations was that two 50 Ohm impedances were added at each port when using the Advanced Design System (ADS) software to simulate the equivalent circuit. On the other hand, the symmetry of the two added impedances at two ports in the ADS simulations could eliminate the influence of the added impedances on the resonance damping simulations. Therefore, the resonance damping in the calculation and simulation results had the same values, and the resonance frequency changes in calculation and simulation results had different values but reflected the same trend. 

## 3. Discussion

For in-liquid QCM applications, the viscous coupling of the contact liquid to the quartz crystal resonator surface resulted in decreases in both the resonant frequency of the QCM and damping of the resonance. Traditional knowledge has suggested that the resonance damping is only caused by the added equivalent resistance $R_{2}$, and the resonant frequency change was only caused by the added equivalent inductance $L_{2}+L_{3}$. However, both the theoretical calculations and simulations proved that the added equivalent resistance $R_{2}$ also caused resonant frequency change. Therefore, the in-liquid QCM resonant frequency change is comprised of two parts: the frequency change $\Delta f_{L}$ caused by the added equivalent motional inductance $L_{2}+L_{3}$; and the frequency change $\Delta f_{R}$ caused by the added resonant motional resistance $R_{2}$. In actual measurements, the measured frequency change was $\Delta f=\Delta f_{L}+\Delta f_{R}$, but the theoretical frequency change as per Equation (5) was just $\Delta f_{L}$ and did not contain $\Delta f_{R}$. Although $\Delta f_{R}$ was smaller than $\Delta f_{L}$ in most applications, the influence of $\Delta f_{R}$ could not be ignored, especially in in-liquid QCM with large motional capacitance, as $\Delta f_{R}$ increased sharply along with increasing $C^{*}$.

**Figure 6:**
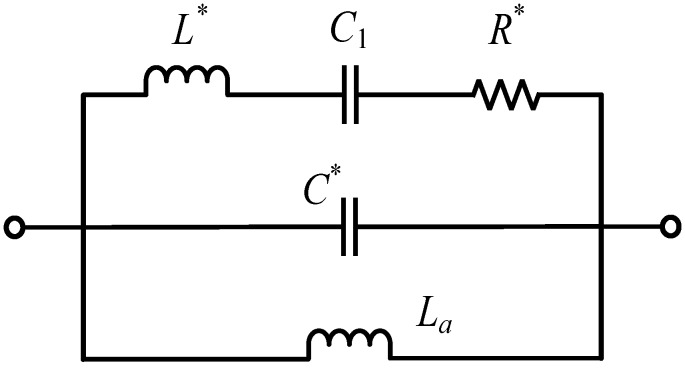
The equivalent circuit for an in-liquid QCM after the addition of inductance $L_{a}$.

Currently, the frequency change $\Delta f_{R}$ is not easily measured accurately, which is an important area of future research. As we could not change the equivalent resistance alone in the actual experiment (the addition of a resistor in series with QCM could not model the increase of equivalent resistance), it was hard to isolate the frequency change $\Delta f_{R}$ caused by the added resonant motional resistance $R_{2}$. Therefore, the existence of unpredictable $\Delta f_{R}$ will lead to errors for in-liquid QCM applications. To resolve these issues, a possible solution was presented: to add an inductance $L_{a}$ (make the resonant frequency of the oscillating circuit that consists of $L_{a}$ and $C^{*}$ equal to f) to eliminate the influence of $R_{2}$ on resonant frequency, as Figure 6 shows.

**Figure 7:**
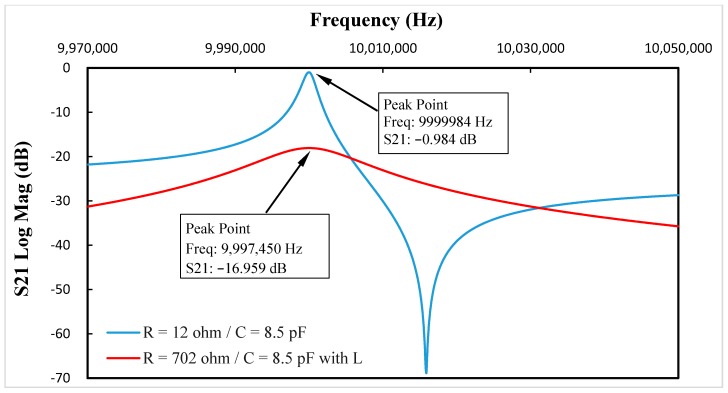
When $C^{*}$ = 8.5 pF, the S21 parameters vs. frequency curve after the addition of inductance $L_{a}$.

Using Group 5 in Table 1 as an example, the S21 parameters vs. frequency curve after added the inductance $L_{a}$ is shown in Figure 7. By comparing Figure 5 and Figure 7, it was clear that the addition of $L_{a}$ with a value of 29.8 µH, the resonance frequency change caused by the added resistance decreased from 2534 Hz to 9 Hz. Furthermore, resonance damping also increased.

## 4. Conclusions

For in-liquid QCM applications, the liquid load was equivalent to a mass loading ($L_{3}$) and a viscous loading ($L_{2}+R_{2}$). The viscous coupling of the contact liquid to the QCM surface led to decreases in resonant frequency and increases in resonance damping. Unlike previous notions, motional resistance caused by viscous loading resulted not only in resonance damping, but also resonance frequency change. Furthermore, the resonant frequency change caused by the added motional resistance increased sharply along with increases in static capacitance. In addition, the resonance damping caused by the added motional resistance decreased along with static capacitance increasing. These results will be instrumental in understanding in-liquid QCM characterization, which is of importance in in-liquid QCM applications.

## Figures and Tables

**Table 1:** The calculated results using the BVD equivalent circuit model.

	$R^{*}\;\left(\Omega \right)$	$L^{*}\;\left(\mathrm{mH}\right)$	$C_{1}\;\left(\mathrm{fF}\right)$	$C^{*}\;\left(\mathrm{pF}\right)$	f (Hz)	$\Delta f_{R}\left(\mathrm{Hz}\right)$	Resonance Damping (dB)
Group 1	12	9.3816	27	0.0	9,999,995	0	–0.984
	702	9.3816	27	0.0	9,999,995		–18.083
Group 2	12	9.3816	27	1.0	9,999,995	262	–0.984
	702	9.3816	27	1.0	9,999,733		–18.065
Group 3	12	9.3816	27	3.5	9,999,995	898	–0.984
	702	9.3816	27	3.5	9,999,097		–17.860
Group 4	12	9.3816	27	6.0	9,999,995	1478	–0.984
	702	9.3816	27	6.0	9,998,517		–17.470
Group 5	12	9.3816	27	8.5	9,999,995	1983	–0.984
	702	9.3816	27	8.5	9,998,012		–16.959
